# Phosphate environment and phosphate uptake studies: past and future

**DOI:** 10.1007/s10265-024-01520-9

**Published:** 2024-03-22

**Authors:** Tetsuro Mimura, Robert Reid

**Affiliations:** 1 Department of Biosciences, Faculty of Bioenvironmental Sciences, Kyoto University of Advanced Science, Kameoka, Kyoto, 621-8555 Japan; 2 College of Bioscience and Biotechnology, National Cheng Kung University, Tainan, 70101 Taiwan; 3 Department of Biology, Graduate School of Science, Kobe University, Hyogo, 657-8501 Japan; 4 The Institute for Sustainable Agro-ecosystem Services, Graduate School of Agricultural and Life Sciences, The University of Tokyo, Nishi-Tokyo, Tokyo, 188-0002 Japan; 5 School of Biological Sciences, Faculty of Sciences, Engineering and Technology, The University of Adelaide, Adelaide, South Australia 5005 Australia

**Keywords:** Phosphate, Phosphorus, Plant, Plasma membrane, Transport, Vacuole

## Abstract

The present review explains briefly the importance of phosphorus in the biological activities and states that the most phosphorus of living organisms is absorbed by plants from the soil. Next, previous studies on the mechanisms of phosphate uptake by plants are reviewed as H^+^-dependent or Na^+^-dependent co-transport systems and the phosphate environment in which plants grow is discussed. The evolution of transporter genes and their regulation mechanisms of expression is discussed in relation to the phosphorus environment.

## Introduction

Phosphorus is one of the most important elements for life on earth, when considering the biological functions of the macromolecules that control genetic information, such as DNA and RNA, the molecules that drive energy metabolism such as ATP, and the membrane lipids that form cells (Mimura 1999).

A detailed discussion of why life utilized phosphorus requires consideration of the physicochemical properties of the phosphorus element. According to this analysis, the following two properties become important (1) phosphate esters of orthophosphoric acid can form a wide variety of molecular bonds, (2) orthophosphoric acid has multiple stable negative charges (Kamerlin 2013; Westheimer 1987).

Arsenic is an element having similar properties, and in the past years, it was reported that certain organisms use arsenic instead of phosphorus, but this has been acknowledged to be wrong (Reaves et al. 2012). The reduction of pentavalent arsenic is much easier than that of pentavalent phosphorus, but the facility with which the redox state of arsenic can change may be disadvantageous. Also, arsenate esters are less stable at room temperature which would make it difficult for arsenic to substitute for phosphorus.

Although phosphorus atoms can exhibit different redox states, there are few reactions in vivo in which changes in the redox state of the phosphorus atom are exploited. Chemical reactions with the ester bond of phosphoric acid are easy, which makes it convenient to use in intermediates involved in energy transfer in many biological reactions.

The energy transfer between metabolites in living organisms can be categorized into two types of reactions: ones involving redox reactions, and others dependent on the ester bonds of phosphoric acid. The importance of both respiration and photosynthesis is linked to their ability to convert the energy obtained from redox reactions into energy stored in the ester bonds of phosphate.

On the other hand, neither phosphorus nor phosphoric acid are used in proteins. Even if certain RNAs have enzymatic activity, they are nevertheless proteins, and phosphoric acid is never used in the main frame of proteins. The reason why phosphate groups are not used in the side chains of amino acids that make up proteins remains a curiosity.

We know well that in protein phosphorylation, phosphate groups are introduced into the side chains of multiple amino acids to alter the structure of proteins. Protein kinases and protein phosphatases play quite important roles that regulate protein function. Therefore, it would seem logical that some of the amino acids that make up proteins would have phosphate groups as the original side chain, but this has not been the case in nature. One reason may be that the molecular weight of the phosphate group is too large and that the structural constraints are too strong, since it brings a large number of charges. This may have had a major influence on the way life has evolved.

## Phosphorus content of living organisms

The amount of phosphorus in the plant body is estimated to be about 60 µg per gram of dry weight, or about 2 g per kilogram (Epstein 1956; Marschner 2012). It is the eighth most abundant element in plants. In humans, about 1% of body weight is phosphorus, making it the sixth most abundant element in the body. The reason why it is considerably more abundant than in plants is that bones are made of calcium phosphate.

Most of the phosphorus of living organisms is likely to have originated from phosphorus taken up by plants from the soil. Just as carbon, nitrogen, and sulfur are incorporated into organic matter through plant assimilation, which in turn compose heterotrophic organisms, such as animals, the same thing happens with phosphorus.

**Fig. 1:**
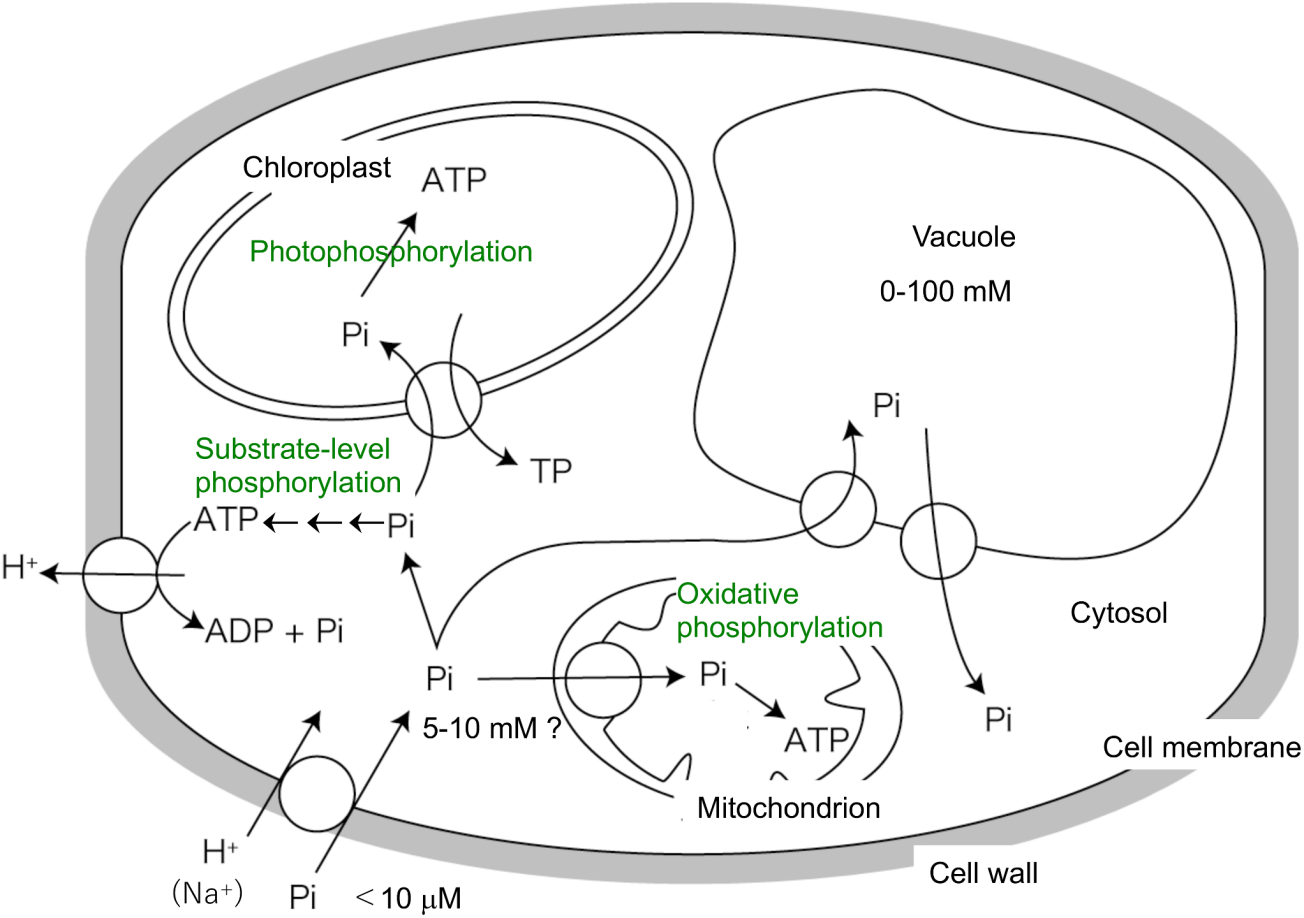
Schematic diagram of orthophosphoric acid (Pi) uptake and incorporation into organic substances in plant cell

However, the major difference between phosphorus and these inorganic elements such as carbon, nitrogen, and sulfur, is that even heterotrophic organisms incorporate inorganic orthophosphate into organic matter in the form of ATP. Phosphorus always enters the cell as the orthophosphate molecule, and its molecular form almost never changes even when it is incorporated into organic matter (Fig. 1). In this sense, phosphorus metabolism is very different from the metabolism of carbon, nitrogen, and sulfur. Although the term “phosphorus assimilation” is now often used, it should be understood that it has a very different meaning to the assimilation of carbon, nitrogen, or sulfur.

## How is the phosphorus in the environment on the earth?

Currently, the abundance of phosphorus on the earth is ranked 13th in terms of Clark’s number, which indicates the composition of surface elements of the earth.

The content of phosphorus itself in the biosphere on the earth’s surface is not small. However, the phosphorus available to plants is limited to orthophosphoric acid in the soil; other phosphorus compounds are rarely used.

The phosphorus element itself can take a variety of molecular forms depending on its redox state and chemical bonds, thus the amount of phosphorus on earth is large. However, the only molecular form that is actually necessary for life is orthophosphoric acid, and its amount in soil is not always sufficient. In fact, the phosphate concentration in soil available to plants is only a few µM. In freshwater environments it is even 1/10 of that level and in seawater, the phosphate concentration near the ocean surface, where microalgae grow, is even lower, often less than 0.1 µM (Mimura 1999).

The K_m_ value of phosphate transport activity reported for land plants is close to the phosphate concentration in the soil environment (Hagen and Hopkins 1955; Hagen et al. 1957). K_m_ values for freshwater algae have been reported to be similar (Mimura 1999).

**Fig. 2:**
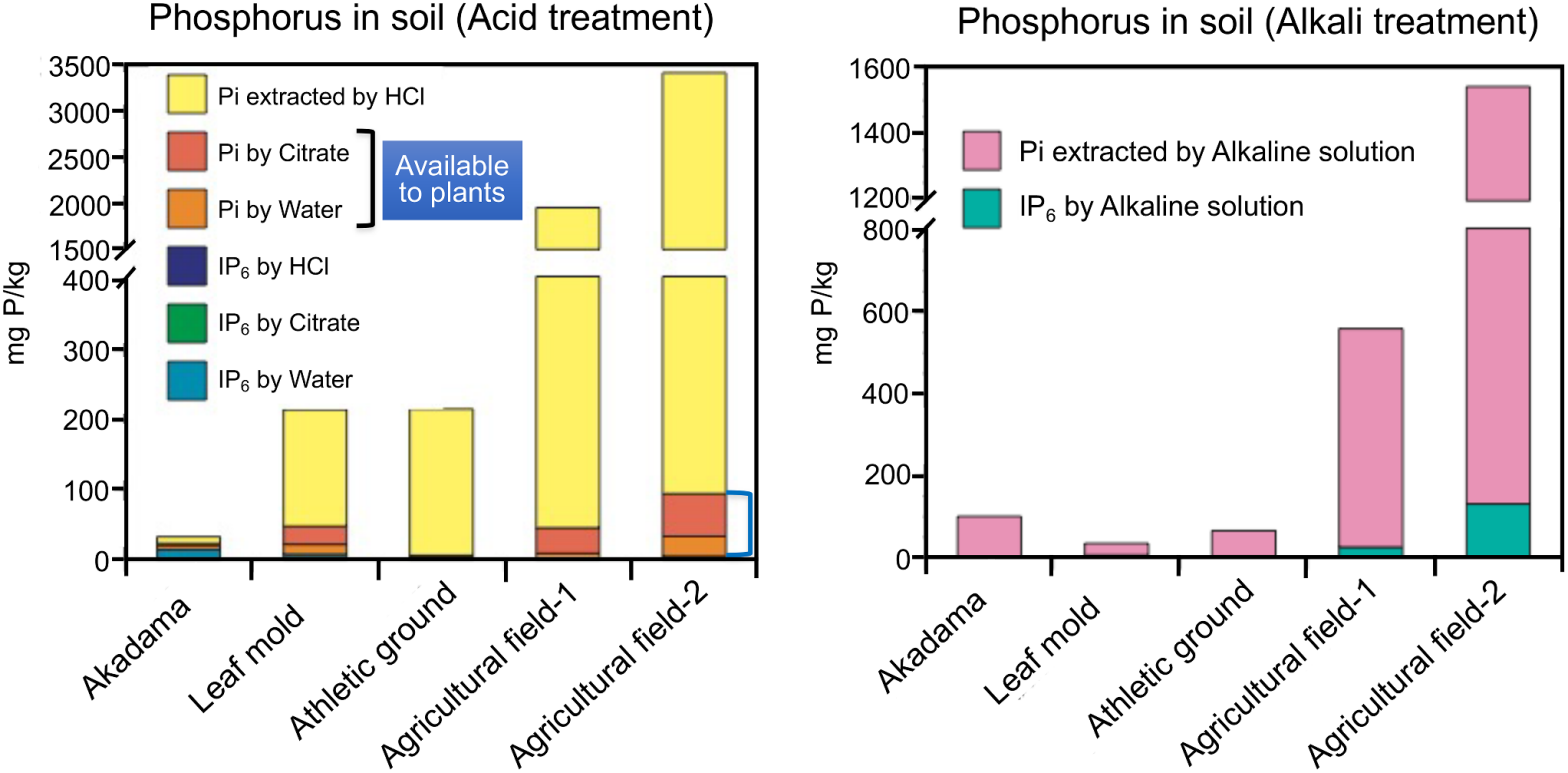
Various forms of phosphorus in soil. **a** Phosphorus extracted from soil in aqueous solution or acidic solutions of HCl or citrate. **b** Phosphorus extracted from soil in alkaline solution. Akadama is a low nutrient loamy soil

Organic phosphates such as nucleic acids and glucose-6-phosphate are present in the soil and are used as a source of phosphate after being degraded by phosphatases secreted by phosphate-deficient plants (Shen et al. 2011; Tran et al. 2010). On the other hand, although myo-inositol hexakis phosphate (IP_6_; phytic acid) is a major organic phosphate in soil (Kurita et al. 2017; Richardson et al. 2001; Turner et al. 2003, 2005), as far as is known, there are no IP_6_ transporters in the plant plasma membrane and plants cannot grow if the only source of phosphorus is IP_6_ (Turner et al. 2002). Liu et al. (2022) discuss strategies to improve IP_6_ availability to crops for future agriculture. Thus, the current phosphorus environment on earth can be considered a phosphorus-deficient environment for land plants. Figure 2 shows the chemical status of phosphorus in various soils in Japan (Fig. 2; the original data was personally supplied from Mr. Yohei Masuda).

## Measurement of phosphate uptake activity and phosphate transport mechanisms

Although the mechanism of the photosynthetic carbon assimilation (Calvin-Benson-Bassam cycle) was largely resolved by tracing the pathway of incorporation of radioisotopic ^14^C into successive carbon compounds in the cycle, over a similar period, various studies on phosphorus uptake, metabolism, and partitioning were conducted using radioisotopic ^32^P (Biddulph 1941; Kamen and Spiegelman 1948; Stou and Hoagland 1939).

Numerous studies have been conducted on the kinetics of phosphate uptake from roots and its distribution in plants (Greenway and Gunn 1966). Studies of phosphate uptake in the eukaryotic cell, yeast, have been useful in elucidating the properties of phosphate uptake in plants. Other studies have revealed different systems for phosphate uptake in prokaryotic bacteria such as *Escherichia coli.* Studies in yeast, as well as in algae and other aquatic plants, have shown that the uptake of inorganic orthophosphate into cells is driven by H^+^-dependent co-transport systems (Goodman and Rothstein 1957; Jeschke and Simonis 1965). Orthophosphate uptake by Na^+^-dependent co-transporters in microalgae (Ullrich and Glaser 1982; Ullrich-Eberius and Simonis 1970), yeast (van Belle and André 2001), and animal cells (Hernando et al. 2021) is also known to exist.

**Fig. 3:**
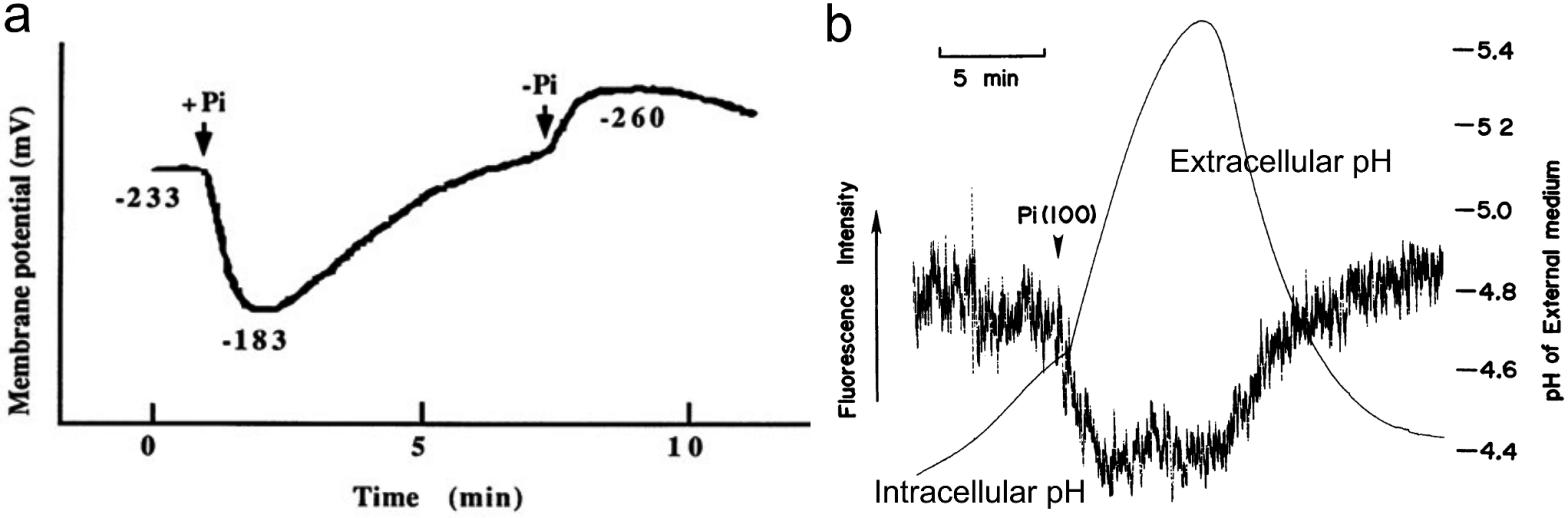
Uptake mechanism of inorganic phosphate by plant cells. **a** Electrophysiological measurement during phosphate uptake of *Lemna gibba* by conventional microelectrode method (cited from Ullrich-Eberius et al. 1981). **b** Measurements of changes in extracellular and intracellular pH during phosphate uptake of suspension cultured cells of *Catharanthus roseus* (cited from Sakano et al. 1992). Extracellular pH was measured by a micro glass pH-electrode and intracellular pH was measured by a pH-dependent fluorescent dye BCECF-AM.

For the discovery of H^+^-dependent phosphate co-transport in plant cells, electrophysiological analysis was complemented with radioisotope studies. It was proposed that a positive charge is brought into the cell during uptake of orthophosphate, since depolarization of the cell membrane occurred when orthophosphate was taken up under acidic conditions. The cell membrane was depolarized by the positive charge carried with phosphate and H^+^s (Fig. 3a) (Ullrich-Eberius et al. 1981).

Furthermore, simultaneous measurements of phosphate uptake and extracellular pH change in suspension-cultured cells of *Catharanthus roseus* suggested that at least 4 H^+^ per 1 molecule of phosphate is transported (Sakano 1990). Furthermore, measurements of intracellular pH using a pH-sensitive fluorescent dye has demonstrated that transient cytoplasmic acidification occurs during this process (Fig. 3b) (Sakano et al. 1992). This acidification is immediately counteracted by cytoplasmic pH homeostasis.

We also found that phosphate transport in the internodal cell of *Chara australis* (the former name of *Chara corallina*) is not driven by H^+^ but is a Na^+^-dependent co-transport system, the same as in microalgae and animal cells (Reid et al. 2000). It is well known that the Characeae are the immediate phylogenetic ancestors of land plants. Here, we estimated that at least 6 Na^+^ are simultaneously transferred for each molecule of phosphate. In seed plants, it has also been reported that phosphate transport in *Zoster marina*, a type of seaweed, is a Na^+^-dependent co-transport (Rubio et al. 2005).

## Phosphate transporter genes

The gene for the plasma membrane-type phosphate transporter was first cloned as PHO84 in yeast (Bunya et al. 1991). This type is now called Pht1. Subsequently, by using a yeast PHO84 sequence and EST data of *Arabidopsis*, two genes of Pht1 from *Arabidopsis thaliana* have been cloned (Muchhal et al. 1996; Smith et al. 1997), and currently a gene of the Pht1 group has also been cloned from various plants. Additions to Pht1 family, PTA- and PTB-type transporters in the plasma membrane, have also been reported in algae and mosses (Jia et al. 2021; Wang et al. 2020) while a SULTR-like phosphorus distribution transporter (SPDT) has been found in rice (Yamaji et al. 2017). The phosphate transporters in the cell membranes of cyanobacteria and eubacteria appear to be quite different from these transporters.

Phosphate transporters from Pht2 to Pht5 are known to exist in organelle membranes, while PHO transporters have been associated with phosphate efflux (Wang et al. 2021a, b). In most cases, the function of the transporter has only been inferred from the analysis of mutants, with only a few reports where the ability to transport phosphate has been confirmed.

We have recently cloned the Pht1 gene from *Chara braunii* and confirmed using *Xenopus* egg expression system that it functions as a Na^+^-dependent co-transporter of phosphate (Mimura et al. in preparation).

## Phosphate condition and gene expression

As mentioned above, in the environment in which plants and other photosynthetic organisms grow, the generally available water-soluble or less-soluble phosphate content is not large. Therefore, agriculture requires the input of large amounts of phosphate fertilizer. In recent years fears have been raised that phosphate fertilizer needed for agriculture may be consumed faster than oil and other energy materials. Ways to avert this potential global shortage have resulted in a renewed focus on the management of phosphate resources (Cho et al. 2021).

We do not know exactly how long the availability of phosphorus in the environment has been low and how this has affected the evolution of different organisms, and the strategies they use to overcome this limitation in supply. It is likely that if most organisms existed in an environment with low concentrations of orthophosphate, phosphate deficiency-inducible (PDI) genes that we now know would be expressed under phosphate deficiency, and in the presence of excess phosphate, such gene expression would be suppressed. The possibility should be considered that the suppression of such gene expression in the presence of excess phosphate may have occurred more recently in evolution. In fact, the presence of the key transcription factor Phr in the genomes of many algae and other organisms known so far may imply that the expression of PDI genes is usual. In addition, the regulatory mechanism of genes related to phosphate transport and metabolism in yeast, the so-called PHO regulon, is very different from that of eukaryotic photosynthetic organisms, indicating that the regulatory system for phosphorus environmental responses has evolved in a markedly different manner from that of similar transporters.

If plant growth in a low-phosphorus environment is general, the question is, how can plants continue to grow in such a low-phosphate environment. It has been shown that growth can continue even at low concentrations as long as the minimum phosphate necessary for growth is continuously supplied from the external environment.

It is also known that the presence of excess phosphate has an inhibitory effect on plant growth (Mimura et al. 1990, Sakano et al. 1995), and that when such an environment is encountered, the phosphate that is first taken up and is not immediately needed for metabolism and other processes, is accumulated in the vacuole. The accumulation in the vacuoles and the resulting phosphate homeostasis in the plant cytoplasm may suggest that plant cells routinely grew in a low-phosphate environment, rather than evolving to adapt to the occasional excess phosphate environment. Under excess phosphate, it would have been important to suppress the expression of transporters in phosphate-rich environments to reduce the uptake of excess phosphate. Further, Arabidopsis mutant of the vacuolar phosphate uptake shows growth-redundancy. This means that if the cytoplasmic phosphate concentrations become too high, growth will be adversely affected (Liu et al. 2016).

There is an interesting story that geologically, Australia is a very old country and has highly weathered soils with very low nutrients, particularly phosphate. This meant that during evolution Australian plants had to obtain phosphorus from very low concentrations. Currently we know many adaptations such as cluster roots, mycorrhizae and non-deciduous plants (dropping leaves risks losing valuable phosphate). But as every Australian gardener knows, native plants are very sensitive to phosphorus toxicity and all garden centers have special fertilizers low in phosphate for such plants.

As mentioned above, the fact that the transcription factor Phr, which is important for PDI gene expression, is present in a wide range of genomes from algae to other organisms, leads us to assume that regulation of PDI gene expression is the ancestral type and that suppression of gene expression by the presence of high levels of phosphate may be a more recent character.

The nature of the adaptation of original horticultural plants to phosphate environments in Australia needs to be examined at the genomic level.

We suspect this sensitivity arose because never experiencing high phosphate conditions, they do not respond to an abundance of phosphate by down-regulating phosphate transporters and so continue to accumulate phosphate till it becomes toxic. Alternatively, it is possible that Australian plants have lost their ability to adapt to excess phosphate for some other reason, something that warrants further study.

We also have to consider the following question, how plants evolved an adaptation system for high phosphate levels in nature. Recently it has been reported that about 800 million years ago, asteroid showers bring much phosphorus from outer space to earth and a great biological breakthrough was brought about (Terada et al. 2020). If this is true, organisms before this astronomical event, had adapted to very low level of phosphate, but afterword they must adapt to the high phosphorus condition in aquatic environments, and then PDI system has evolved. We might call it Phosphate-Increase Induced (PII) system.

This concept may be the same for nitrogen although we do not know from where nitrogen came. It can be assumed that plants originally adapted to low nitrogen levels in the environment had higher activity of nitrogen transporters and more active nitrogen assimilation metabolism, but when nitrogen levels increased, they protected themselves from the toxic environment of high nitrogen by suppressing the various molecular mechanisms that were necessary to adapt to those low nitrogen environments.

Currently, adaptation to a low-nitrogen environment requires that a very small amount of nitrogen (nitrate ions) be present outside the cell, which is why the concept of “Tranceptor” has been proposed and clarified (Liu and Tsai 2003). It is generally understood that the expressions of low-nitrogen-responsive genes are meaningless unless even a small amount of nitrogen is present in the external environment, and if this is the case, a similar situation could be considered for phosphate. Of course, nitrogen can be acquired in different forms (NH_4_, NO_3_, or urea) and the induction mechanism for each may be different, which is different from phosphate, but how the final external nitrogen concentration is integrated as intracellular nitrogen levels is still unknown.

At present, however, we do not know much about how plant cells recognize the level of phosphate in their environment, only that inositol phosphate, which binds to the SPX domain, is important (Jung et al. 2018; Secco et al. 2012). In this case, it must be assumed that the intracellular phosphate concentration is proportional to inositol phosphate, but it is not yet clear whether the sensor for the cytosolic phosphate level is an enzyme related to inositol phosphate biosynthesis or something else.

**Fig. 4:**
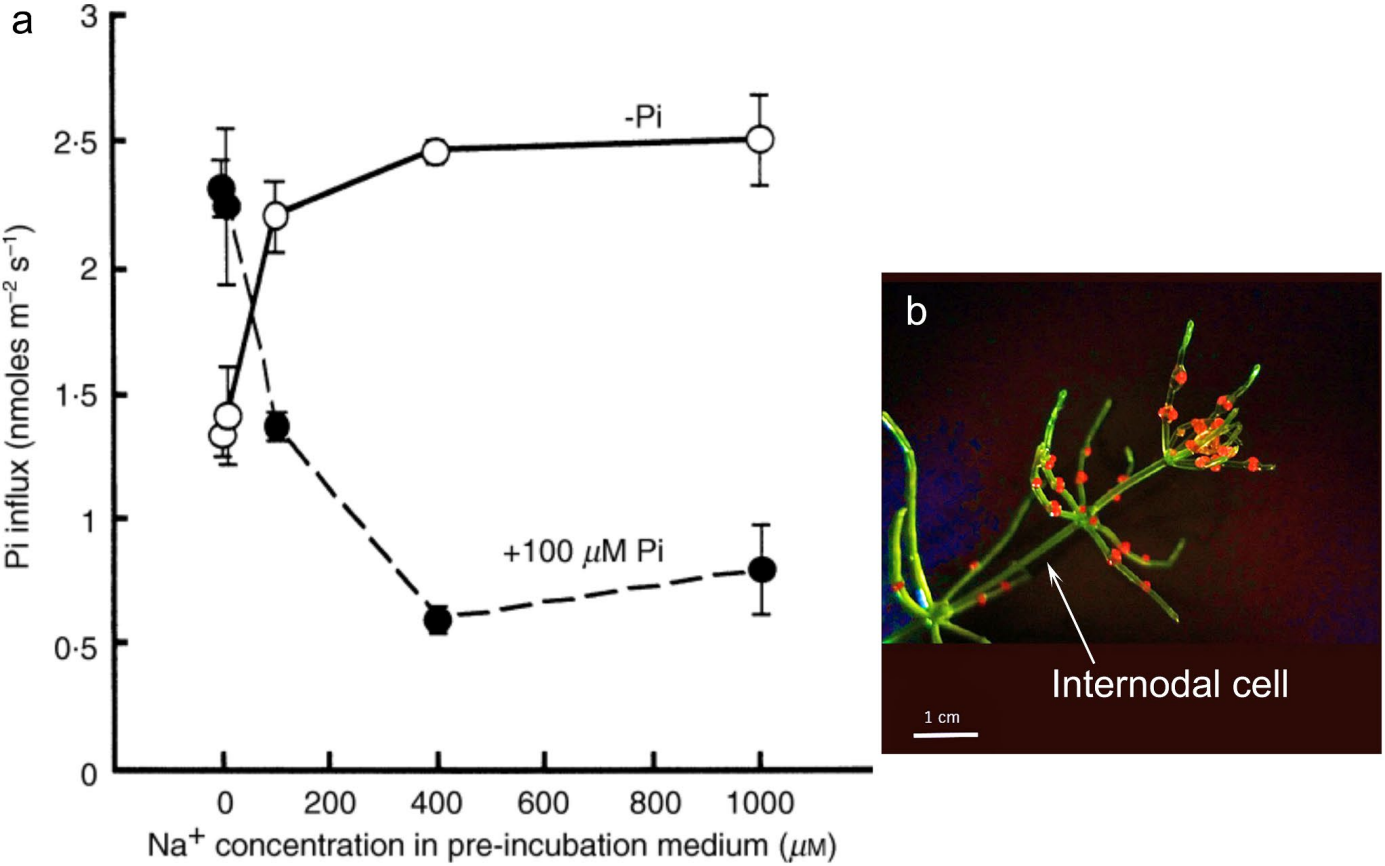
**a** Effect of Na^+^ concentration in the pre-treatment bathing medium in the absence (open circle) or presence (closed circle) of Pi on induction of Na^+^-dependent Pi influx of *Chara australis*. Cells were pre-incubated in 0·1 mM KCl, 0·5 mM CaCl_2_, various concentrations of NaCl with and without 100 µM Pi for 6 d (cited from Mimura et al. 2002). **b** A photograph of *Chara australis* supplied by Ms. Hitomi Matsumoto. The internodal cell indicated is a single cell whose length is about 3 cm

Finally, we would like to discuss the data we have obtained in the *Chara* internodal cell (Fig. 4a, b).

As mentioned above, *Chara* internodal cells exhibit increased phosphate transport activity at the single-cell level in response to a phosphorus-deficient environment (Mimura et al. 1998). Since this transport activity has been demonstrated to be a Na^+^-dependent transport system (Mimura et al. 2002; Reid et al. 2000), a phosphate-depleted condition of the cell interior can be created by removing Na^+^ from the external medium, even in the presence of phosphate. Such internal phosphate deficiency in the presence of external phosphate, is not induced in organisms that utilize the H^+^-dependent transport system.

When we looked at how phosphate transport activity changed in this case, we found that in an environment with sufficient Na^+^, the capacity to transport phosphate increased under phosphate deficiency. In contrast to deficiency, when there is sufficient phosphate, phosphate transport activity remains low. This is the same as the general phosphate environmental response. However, when a cell was pretreated for one week without Na^+^ in the external solution but in the presence of phosphate, phosphate uptake activity was greater in cells exposed to an environment with sufficient phosphate than in cells exposed to a phosphate-deficient environment (Fig. 4a) (Mimura et al. 2002). This cannot be explained simply by a decrease in the intracellular phosphate concentration and the response of the phosphate transport system to it. This may suggest that a more complex regulatory system is hidden in the phosphate deficiency response, at least in the *Chara* internodal cell.

In the above, we have simply reviewed the history of research on the phosphate transport mechanism of plants, mainly uptake from the external environment, and the phosphate response to the environment, as well as the issues that still need to be clarified. We eagerly await the results of further research into this fascinating area of plant nutrition.
